# Uncommon Finding of a Soft Palate Schwannoma: A Case Report

**DOI:** 10.7759/cureus.50172

**Published:** 2023-12-08

**Authors:** Khulood A Siddig, Omar M Nazhat, Nadia M Saleem, Iyad S Hamadi, Fatma A AlHashimi

**Affiliations:** 1 Department of Otorhinolaryngology, Dubai Academic Health Corporation, Dubai, ARE; 2 Department of Otorhinolaryngology, Dubai Hospital, Dubai, ARE; 3 Department of Medicine, Dubai Academic Health Corporation, Dubai, ARE

**Keywords:** schwann cells, perineural tumor, soft palate, oral cavity, schwannoma

## Abstract

Schwannomas are relatively slow-growing benign tumors of the nerve sheath. Approximately 25-40% of schwannomas occur in the head and neck region. However, schwannomas that present in the oral cavity are relatively rare, constituting around 1% of all described cases in the head and neck region. We report a case of a 20-year-old female who was found to have an intraoral palatal schwannoma. The patient presented with a painless swelling located on the right side of the soft palate. Investigations and management were commenced, and a computed tomography (CT) scan with contrast was done, which revealed cystic changes in a large soft palate mass lesion with a heterogeneous enhancement. The mass was surgically excised and sent for histopathological examination. The diagnosis of schwannoma was made due to the presence of the characteristic Antoni A and Antoni B areas. The immunohistochemical study done was positive for protein S-100. The postoperative follow-up went uneventful.

## Introduction

Schwannomas are rare, encapsulated, and benign perineural tumors that arise from Schwann cells of the peripheral, cranial, or autonomic nerves [1,2]. Although they have been reported in all ages, schwannomas generally present during the second or third decades of life [1,3]. The head as well as neck regions are the most commonly affected areas for this tumor, where 25-40% of the cases have been reported to occur, and the intraoral location represents only 1% of these cases [4-6]. Imaging is helpful as the initial workup [3]. Treatment of these tumors is generally by complete surgical excision and recurrence is low after full removal [6].

## Case presentation

**Figure 1:**
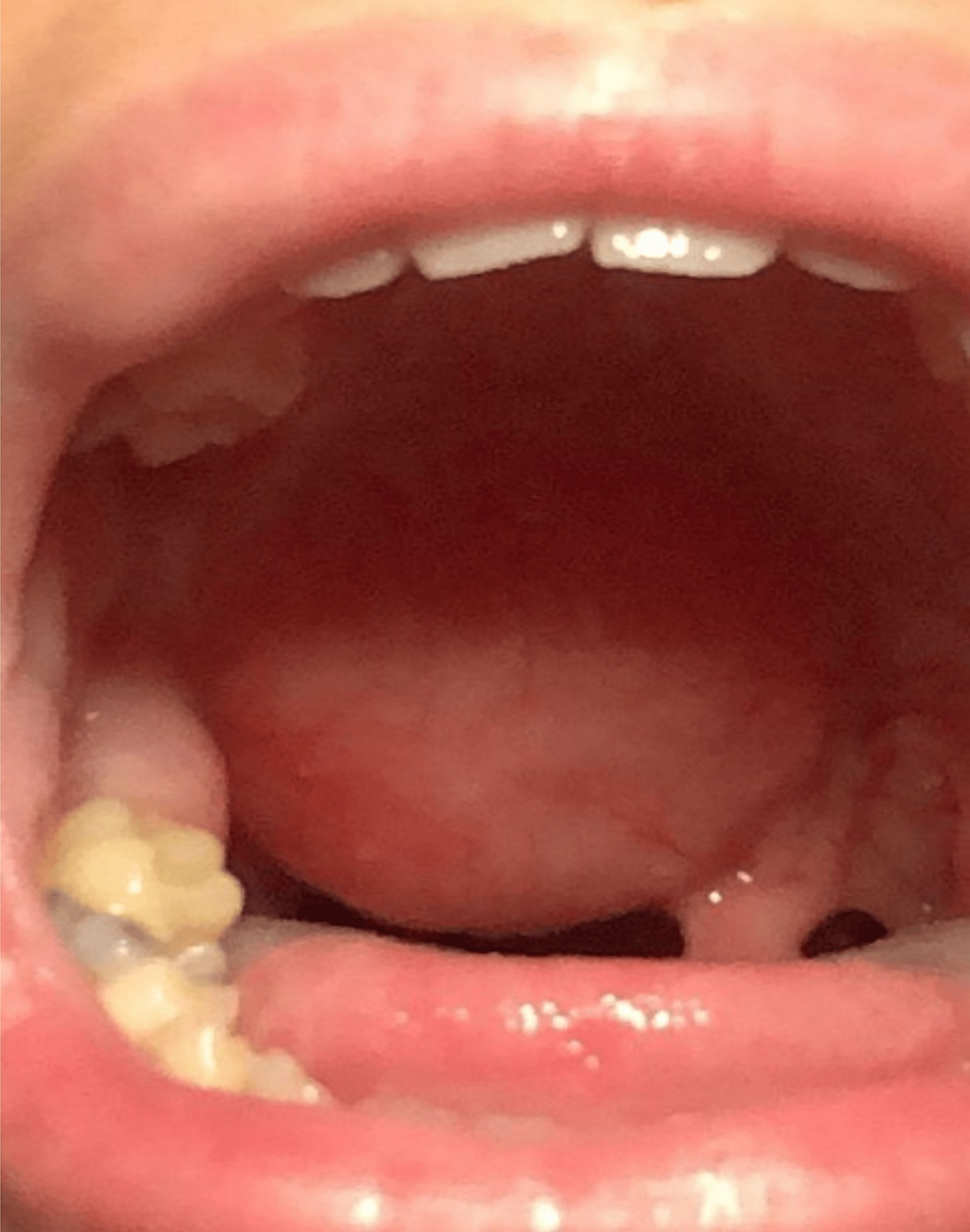
Preoperative photograph of the mass involving the right of the soft palate

A 20-year-old female presented to our clinic with a gradually increasing, big, cystic, painless swelling in the right aspect of the soft palate (Figure 1), which was first noted five years ago.

Her condition started as a foreign body sensation in the throat, which progressed to swallowing difficulty and a change of voice as the swelling increased in size. She had no complaint of breathing difficulty and was otherwise a healthy individual without any history of smoking or alcohol consumption. Clinical examination of the oral cavity revealed a 3x3 cm solitary, cystic, smooth-surfaced mass nearly involving the whole right aspect of the soft palate. There was no tenderness to palpation and no evidence of paraesthesia. The overlying mucosa was normal without any ulceration, and the rest of the oral cavity was normal. No palpable lymph nodes were found at any of the head and neck regions.

**Figure 2:**
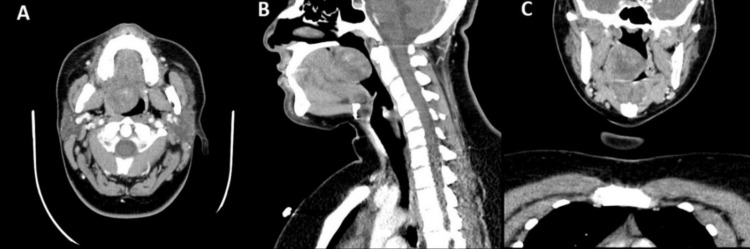
A: Axial, B: Sagital, C: Coronal cuts of the computed tomography (CT) scan with contrast showed a large soft palate mass lesion with heterogeneous enhancement narrowing the airway

The patient’s general laboratory blood reports were unremarkable. A computed tomography (CT) scan with contrast was done and revealed cystic changes in a large soft palate mass lesion with a heterogeneous enhancement measuring about 4 X 3 cm, causing narrowing of the airway (Figure 2).

A provisional differential diagnosis of a minor salivary gland tumor, massive retention cyst of the soft palate, and fibroma was assumed. However, there was no suspicion regarding schwannoma because of its rarity in this location.

To establish the diagnosis, the patient was planned for an excisional biopsy via a transpalatal approach under general anesthesia. The procedure was explained to the patient and written consent was obtained.

The patient was taken for surgery with the usual preoperative preparation. Total excision of the mass was performed via a horizontal incision parallel to the fibers of the palatal musculature. A blunt dissection was carried out away from the posterior soft palate and the palatal muscles, preserving the muscles and mucosa. The mass was found to be round, regular, and cystic in nature with a clear capsule. It was excised entirely with an intact capsule. Closure of the incision was then done, and the procedure was completed smoothly without any complications.

The postoperative period was uneventful, and the patient was treated with antibiotics and anti-inflammatory drugs and advised to maintain good oral hygiene.

**Figure 3:**
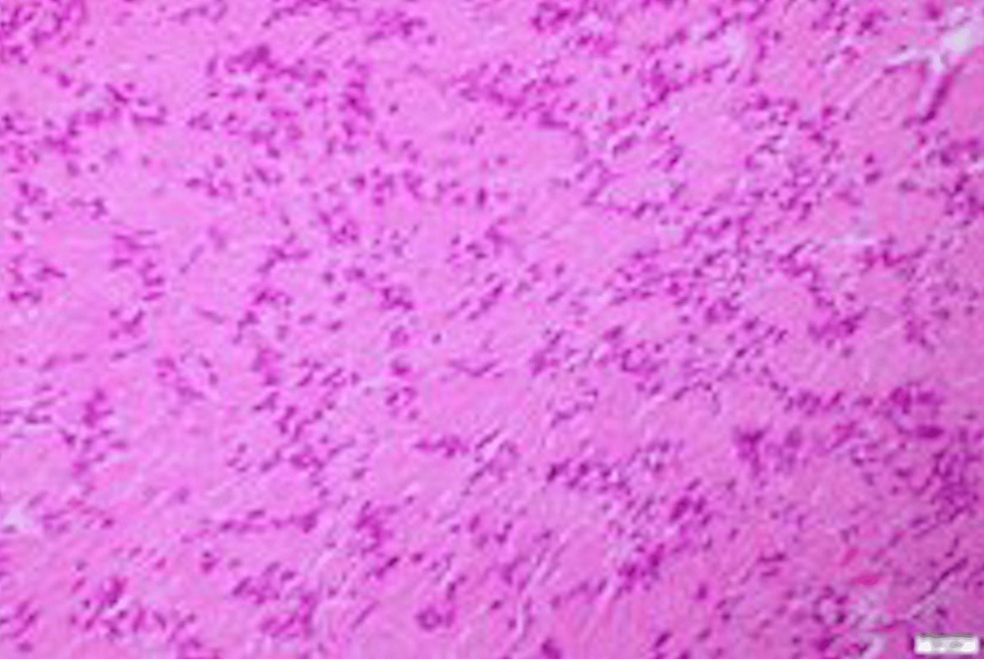
Histological picture of the lesion showing Antoni A area with nuclear palisading (H&E stain 200x)

**Figure 4:**
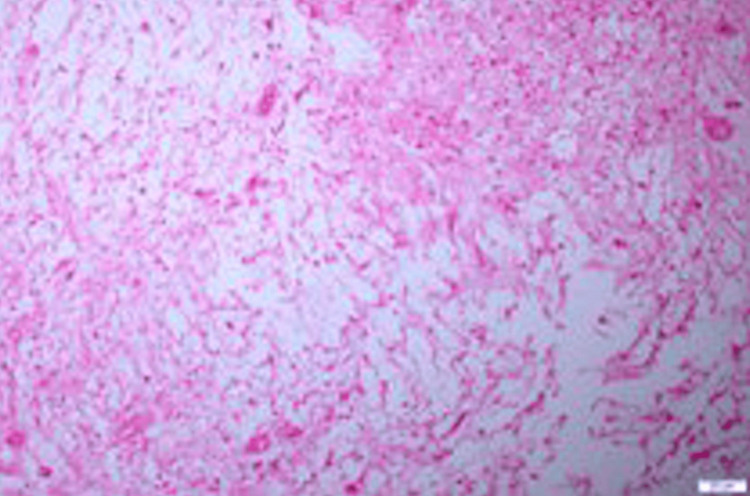
Histological picture of the lesion showing the Antoni B area with less cellular, loose matrix, mild inflammatory cells, and delicate collagen fibers (H&E stain 200x)

**Figure 5:**
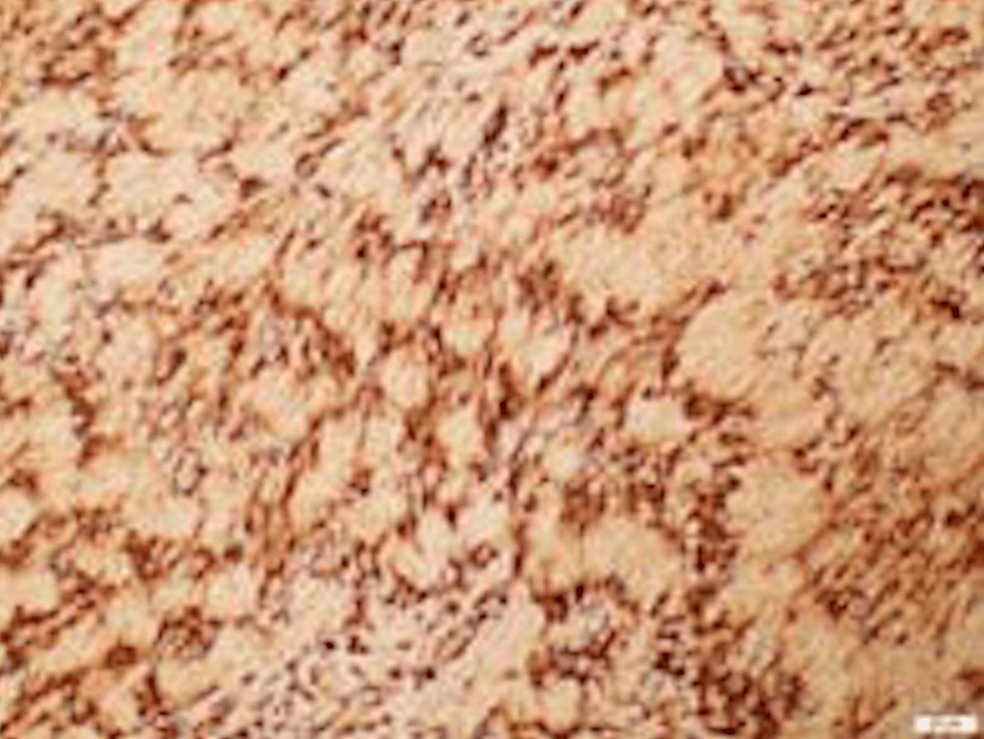
Histological picture of the lesion showing S100 immunomarker positive in Schwann cells (200x)

Histopathological examination revealed the presence of characteristic Antoni A and Antoni B areas and the immunohistochemical study was positive for protein S-100 (Figures 3-5), which confirmed the diagnosis of schwannoma.

At her follow-up visit to the clinic, the surgical site was healing well and after 18 months, she was completely asymptomatic with normal mucosa and no residual palatal dysfunction or signs of tumor recurrence.

## Discussion

It was as early as 1908 that schwannomas were described by Verocay [7]. It was theorized later on, in the year 1935, that the origin of these tumors was from nerve sheath elements, thus leading to the coining of the term neurilemmomas [1]. Locating the exact nerve from which they originate is near impossible, even though they are known to originate from nervous tissue [8].

Intraoral schwannomas are mainly located in the tongue, and the tip is generally the least affected area. They can also be located in other areas like the palate, buccal mucosa, gingiva, base of the mouth, buccal mucosa, or lips, but these are less common [4,6]. Leu and Chang, after reviewing 52 head and neck schwannoma cases over a span of eight years, found that seven cases reported schwannomas of the oral cavity, one case each in the hard and soft palate, two cases in the submasseteric area, and one case in the lower lip and on in the tongue [9].

Schwannomas are slow-growing tumors that usually present as well-encapsulated, solitary lesions and are firm in consistency [3]. In our case, the patient presented with a solitary, solid, painless, and smooth surfaced swelling. The pressure effect of the tumor onto the adjacent nerve may cause paresthesia [10]. However, in our case, the patient did not report any pain or paresthesia.

Additionally, tumors that arise from small nerves are found to be freely mobile, in contrast to ones arising from larger nerves. They can only move along the nerve’s axis [8]. Notably, since the cranial nerves I (optic nerve) and II (olfactory nerve) lack Schwann cells, schwannomas cannot originate from these nerves [5].

Differential diagnoses for schwannomas found in the oral cavity include pleomorphic adenoma or lipoma, especially due to their slow-growing nature and the lack of neural symptoms. Other differentials to be considered are minor salivary gland tumors, fibroma/papilloma, mucous retention cysts, palatal abscess, squamous cell carcinoma, and lymphoma [4].

The microscopic features of these tumors are important to differentiate them from other pathologies. There are two main characteristic types. The first one is called Antoni A. These consist of aligned fusiform cells, arranged to form a typical palisading structure, between the small eosinophilic masses and fibrils forming Verocay bodies. The second type, called Antoni B, is made of a smaller number of cells. The spindle cells in Antoni B are arranged randomly within a loose myxomatous stroma [5].

Other tests that aid in making the diagnosis of schwannoma include immunohistochemical tests, namely, the S-100 protein (peripheral nerve sheath neoplasm marker) [4,6].

Intraosseous schwannomas are normally seen in the posterior part of the mandible. They appear on radiographs either as multilocular or unilocular radiolucencies. Intrabony tumors are generally not associated with paresthesia or pain [4]. Schwannomas may also lead to secondary erosion of the mandible. In this case, the tumor did not cause pain and on the panoramic radiograph, it had a unilocular radiolucency [8]. Excision of the tumor was done under local anesthesia. Histopathological tests later confirmed the diagnosis. Nonetheless, our case showed soft tissue lesions in the soft palate without any signs of bone erosion.

Imaging such as computed tomography (CT) and magnetic resonance imaging (MRI) can be done not only to understand the extent of the lesion but also to help with determining the list of differential diagnoses [3]. The CT scan of schwannomas shows well-circumscribed, homogenous masses of soft-tissue density [11]. The diagnosis, however, is confirmed by histopathological examination [3].

Furthermore, in another study describing two cases of intraoral schwannomas, one of the cases was that of schwannoma at the base of the tongue, with contrast-enhanced MRI done showing a well-defined enhancing mass. In the other case, the schwannoma was located on the floor of the mouth and a CT scan with contrast was done, which showed a well-defined, heterogeneously enhancing lesion. Histopathologic tests confirmed the diagnosis is both cases [12]. Similarly, in our case, the preoperative CT scan finding was also a well-defined heterogeneous enhancement mass (Figure 2).

In a systematic review consisting of 45 cases of palatal schwannomas, complete surgical removal was concluded to be the best treatment option [3]. The malignant transformation of schwannomas is still controversial, with only a few cases reported. Generally, the prognosis is good [13].

## Conclusions

Schwannomas are benign perineural tumors originating from Schwann cells of the nerve sheath. These lesions rarely present in the intraoral cavity. Imaging can help in demarcating a deferential diagnosis, but confirmatory diagnosis can only be achieved by histopathological examination and immunohistochemical analysis. Surgical excision is the treatment of choice, and recurrence and malignant transformation are extremely rare.
